# Circ_0001671 regulates prostate cancer progression through miR-27b-3p/BLM axis

**DOI:** 10.1038/s41598-024-63068-x

**Published:** 2024-05-28

**Authors:** Lihong Yang, Yong Ruan, Bin Chen, Yuhang Zhu, Houqiang Xu

**Affiliations:** 1 Key Laboratory of Animal Genetics, Breeding and Reproduction in the Plateau Mountainous Region, Ministry of Education, College of Life Sciences, Guizhou University, Guiyang, 550025 China; 2 College of Animal Science, Guizhou University, Guiyang, 550025 China

**Keywords:** Prostate cancer, miR-27b-3p, circRNA, BLM, Progression, Cancer, Cell biology

## Abstract

Prostate cancer (PCa) ranks as the second most prevalent cancer among males globally. However, the exact mechanisms underlying its progression remain inadequately elucidated. The present study sought to investigate the role and underlying molecular mechanism of hsa_circ_0001671 (circ_0001671) in the pathogenic behavior of PCa cells. Guided by the ceRNA theory, miR-27b-3p was employed to identify circRNAs that could potentially regulate Bloom Syndrome Protein (BLM). A series of experimental approaches including bioinformatics, luciferase assays, Fluorescent In Situ Hybridization (FISH), RNA-pulldown, and RNA Immunoprecipitation (RIP) were utilized to validate the miRNA sponge function of circ_0001671. Divergent primer PCR, RNase R treatments, and Sanger sequencing were conducted for the identification of circ_0001671. Quantitative RT-PCR and Western blot analyses were performed to validate gene expression levels. Both in vitro and in vivo experiments were conducted to assess the functional role of circ_0001671 in PCa cells.It was observed that the expression levels of circ_0001671 and BLM were significantly elevated in PCa tissues and cell lines, whereas miR-27b-3p showed decreased expression. Circ_0001671 was found to promote cellular proliferation, migration, and invasion, while inhibiting apoptosis. In vivo assays confirmed that circ_0001671 facilitated tumor growth. Further mechanistic studies revealed that circ_0001671 acted as a competing endogenous RNA (ceRNA) for BLM by sponging miR-27b-3p. The oncogenic role of circ_0001671 in PCa was shown to be modulated through the miR-27b-3p/BLM axis. In conclusion, circ_0001671 exerts an oncogenic effect in prostate cancer through the regulation of BLM by sponging miR-27b-3p, thus suggesting a novel molecular target for the treatment of PCa.

## Introduction

Prostate cancer (PCa) ranks as the second most prevalent malignancy among males globally^1^. The incidence and mortality rates associated with this cancer type are on an upward trajectory, influenced by factors such as dietary habits and lifestyle^2,3^. BLM helicase (BLM), a constituent of the RecQ family of DNA helicases, is implicated in DNA metabolism, genomic stability, and DNA damage responses (Wietmarschen et al.^4–6^). Deletion of BLM is known to precipitate Bloom Syndrome, in which patients exhibit genomic instability and an increased susceptibility to various cancers including prostate, lung, and breast cancer^7,8^. Although BLM has been identified as a potential drug target in cancer therapy, its molecular regulatory mechanisms remain inadequately elucidated.

Circular RNAs (circRNAs) are endogenous non-coding RNAs characterized by their covalently closed-loop structure, devoid of 5' and 3' polarity or polyA tails. This unique structure confers resistance to RNA exonuclease degradation, thereby ensuring their cellular stability^9–11^. Given their stability, abundance, and evolutionary conservation, circRNAs serve as long-lasting regulators of cellular behavior and potent biomarkers (Ju et al.^12^, Chen^13^). Aberrant expression of circRNAs in various cancer cells has been closely associated with the initiation and progression of malignancies^5,6,14^, Previous studies have demonstrated that circRNAs act as microRNA (miRNA) sponges^15,16^, RNA-binding proteins^17^, and regulators of gene transcription and expression (Ashwal et al.^18,19^). Among these functionalities, the miRNA sponge action of circRNAs is the most commonly observed in cancer cells^20–22^. Therefore, understanding the role of circRNAs and their potential miRNA regulators is crucial for elucidating the molecular mechanisms underlying PCa and identifying novel therapeutic targets.

Prior research has identified elevated levels of BLM helicase expression in prostate cancer, indicating that its overexpression can facilitate the progression of this malignancy^23,24^. Additionally, it has been shown that miR-27b-3p can directly target the 3' UTR of BLM, thereby inhibiting its expression and influencing the progression of prostate cancer^8,25^. To date, there has been no documentation of circRNAs that regulate the initiation or progression of prostate cancer through the miR-27b-3p/BLM axis, and the underlying mechanisms remain undefined. In this context, the present study employed the ceRNA theory to screen and identify a circular RNA, circ_0001671, derived from the EIF3B gene, using miR-27b-3p as a linkage. Elevated expression of circ_0001671 was observed in prostate cancer tissues. A series of in vitro and in vivo experiments further corroborated that circ_0001671 is positively associated with the onset of prostate cancer. Mechanistically, circ_0001671 functions as a sponge for miR-27b-3p, thereby upregulating BLM expression by attenuating miR-27b-3p activity and consequently facilitating the progression of prostate cancer. Based on these observations, circ_0001671 holds promise as both a diagnostic biomarker and a therapeutic target for prostate cancer.

## Results

### Characterization and expression analysis of circ_0001671

**Figure 1:**
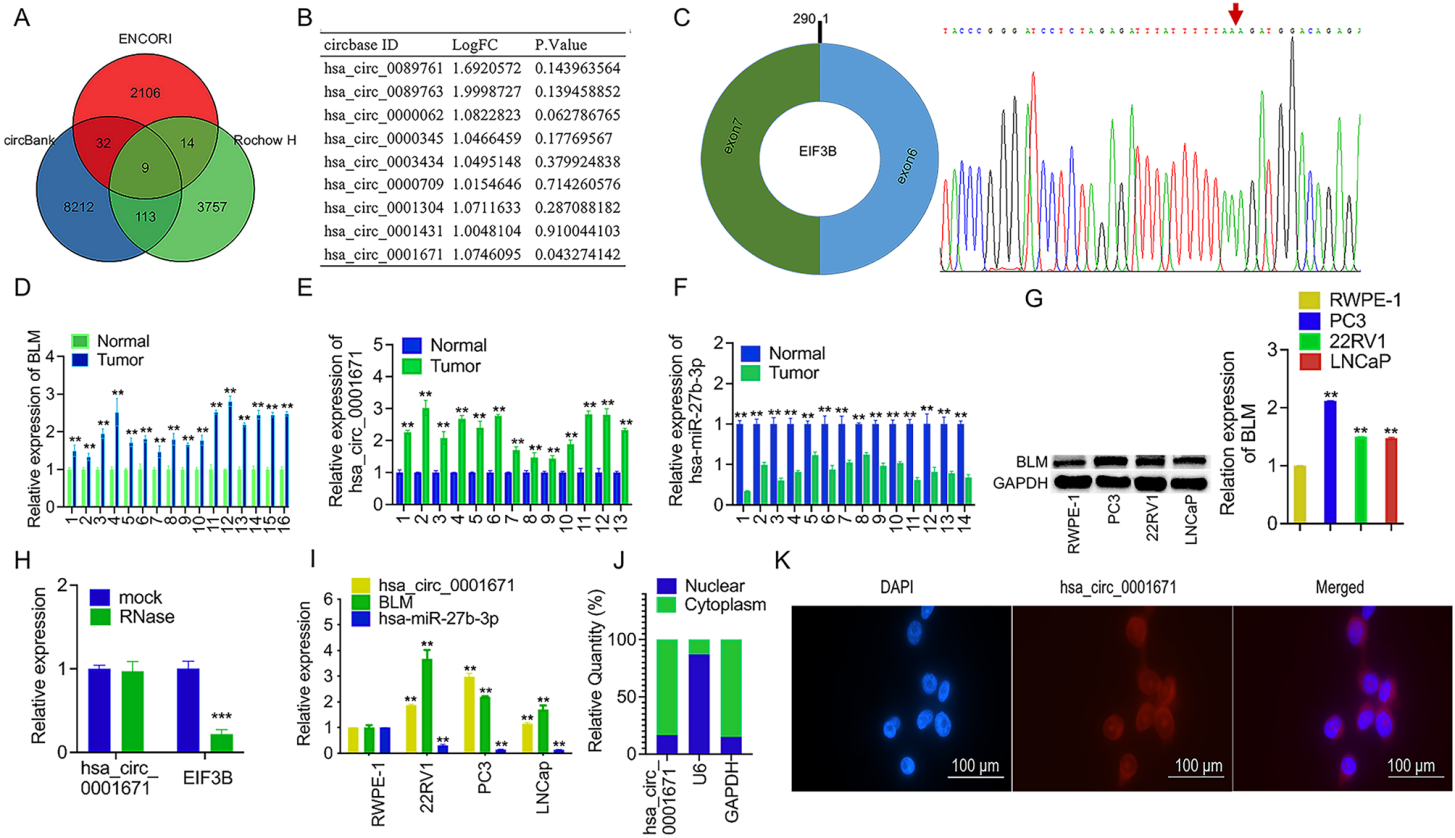
CircRNA Screening and Characterization of circ_0001671. (**A**) circRNAs screening**. **(**B**) Fold change of 9 candidate genes. (**C**) Genomic position of the EIF3B and circ_0001671. The red arrow is the back-splicing from exon 6 to exon 7 of circ_0001671 confirmed by Sanger sequencing. (**D**) BLM expression between 16 PCa tumor tissues and adjacent non-tumor tissues was verified by qPCR. (**E**) The expression of circ_0001671 in 13 PCa tumor tissues and adjacent non-tumor tissues was verified by qPCR. (**F**) Validation of miR-27b-3p expression in 14 PCa tumor tissues and adjacent non-tumor tissues was verified by qPCR. (**G**) Western blot of BLM expression profiles in prostate cancer cell lines (PC3, 22RV1, and LNCaP) and normal human RWPE-1 prostate epithelial cells. (**H**) qPCR analysis of circ_0001671 and EIF3B linear mRNA with RNase R treatment or without. (**I**) Expression profile of BLM, circ_0001671, and miR-27b-3p in prostate cancer cell lines (PC3, 22RV1, and LNCaP) and human normal prostate epithelial RWPE-1 cells by qPCR. (**J**) Results of cytoplasmic and nuclear mRNA isolation experiment. GADPH is a cytoplasmic location marker and U6 is a nuclear location marker. (**K**) Representative images of FISH-circ_0001671 staining in PC3 cells. Probe circ_0001671 was identified as Cy3 (red); Nuclei were stained with DAPI (blue). Scale bars, 100 μm. *p < 0.05, **p < 0.01 by Student’s t-test. Error bars represent SD.

Based on miR-27b-3p data and a pertinent article, nine circRNAs were identified as significantly upregulated (log2(fold change) > 1) in prostate cancer (PCa) (Fig. 1A). Among them, only circ_0001671 exhibited significant upregulation in PCa with a *P*-value < 0.05 (Fig. 1B). The molecule was identified as being spliced from the EIF3B gene, located at chr7:2,404,006–2,406,083, with a final length of 290 nt (Fig. 1C). Divergent and convergent primers were designed to verify the existence of circular transcripts and to confirm circ_0001671 junctions along with linear transcripts (Fig. 1C). These findings confirm the presence of circ_0001671 in cells and its specific amplification by RT-PCR.

It was observed that circ_0001671 is resistant to digestion by ribonuclease R (RNase R), as opposed to linear EIF3B mRNA (Fig. 1H). Quantitative real-time PCR and fluorescent in situ hybridization (FISH) analyses demonstrated that circ_0001671 is predominantly expressed in the cytoplasm of PC3 cells (Fig. 1J, K).

Expression patterns of circ_0001671, BLM, and miR-27b-3p in PCa tumor tissue and adjacent normal tissue were subsequently assessed by quantitative PCR. Compared to the control group, the expression levels of circ_0001671 and BLM were found to be significantly elevated, while miR-27b-3p showed an inverse trend (Fig. 1D–F). In various cell lines, expression levels of circ_0001671 and BLM were notably higher in PC3, 22RV1, and LNCaP cells compared to RWPE-1 cells. Conversely, miR-27b-3p expression was lower in PC3, 22RV1, and LNCaP cells than in RWPE-1 cells (Fig. 1G, I).

### Circ_0001671 upregulates BLM expression

**Figure 2:**
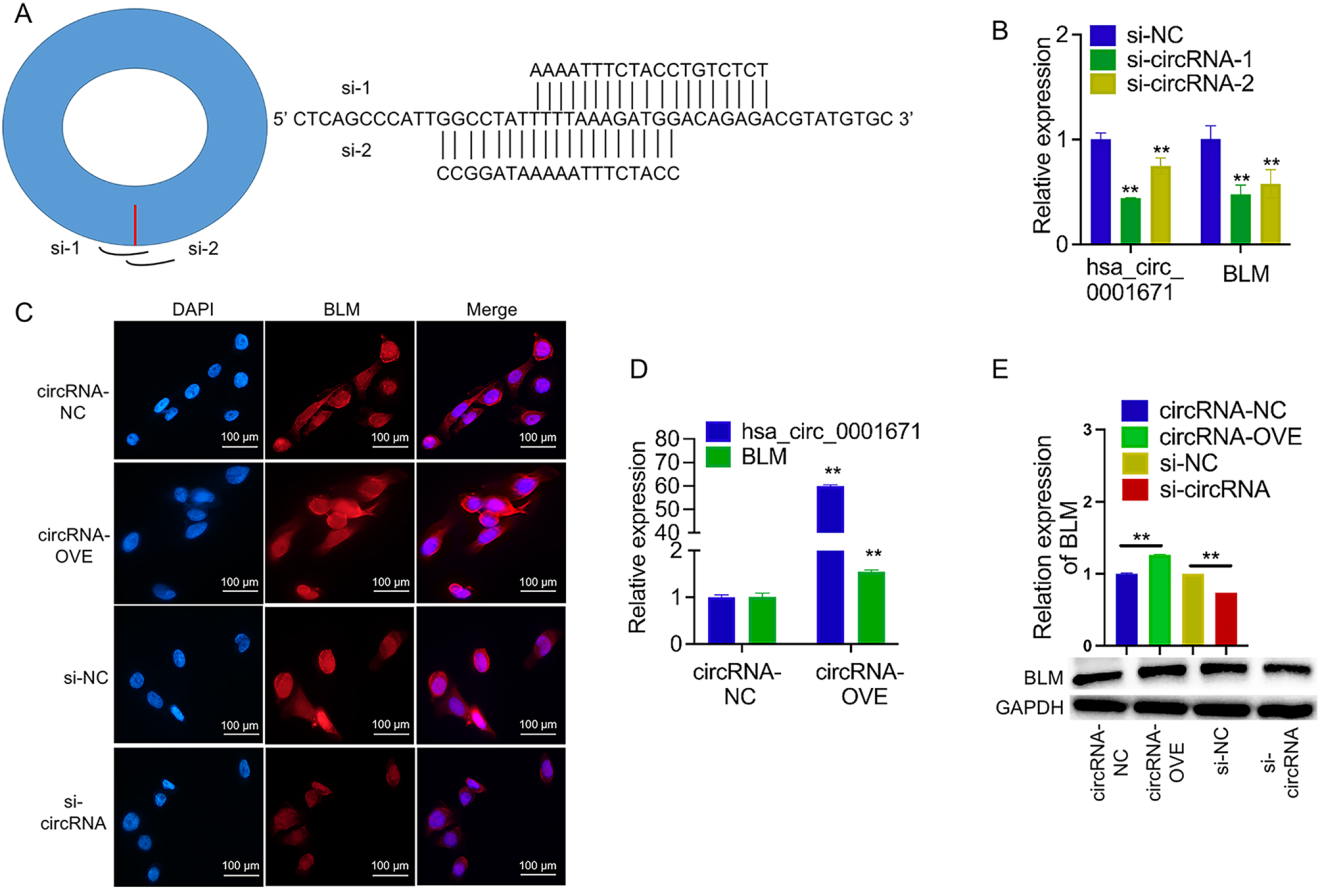
hsa_circ_0001671 promotes the expression of BLM. (**A**) Schematic diagram of the siRNA structure. (**B**) and (**D**) qRT-PCR analysis of hsa_circ_0001671 and BLM RNA expression of hsa_circ_0001671 knockdown and overexpression in PC3 cell lines, compared to negative control siRNA (siNC) and vector control, respectively. (**C**) The effect of hsa_circ_0001671 knockdown or overexpression on BLM was assessed by immunofluorescence staining. (**E**) Western blot analysis of BLM expression when hsa_circ_0001671 knockdown or overexpression in PC3 cell lines, compared to the control, respectively.

To elucidate the influence of circ_0001671 on BLM expression, a circ_0001671 overexpression vector was constructed and short interfering RNAs (siRNAs) were designed. These siRNAs, namely si-1 and si-2, targeted the back-spliced junction site of circ_0001671 to downregulate its expression (Fig. 2A, B). Subsequent qPCR, immunofluorescence, and western blot analyses indicated that overexpression of circ_0001671 positively correlated with increased BLM expression, whereas downregulation of circ_0001671 led to decreased BLM expression (Fig. 2C–E).

### Circ_0001671 promotes PC3 cell proliferation

**Figure 3:**
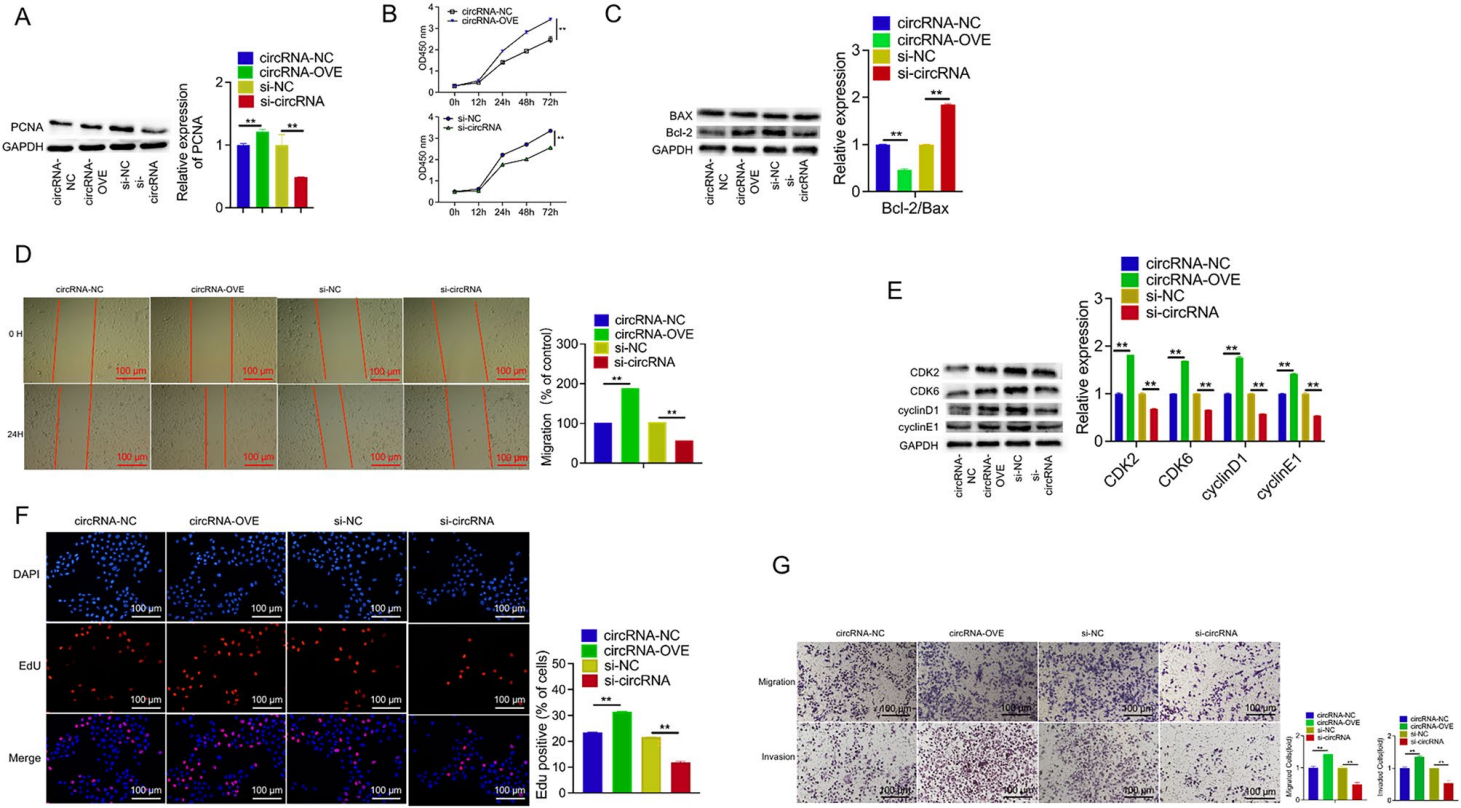
Circ_0001671 promotes PCa Cells proliferation. (**A**) Western blot demonstration of the effect of circ_0001671 knockdown or overexpression on proliferation-related genes. (**B**) CCK8 was assessed for the effect of circ_0001671 knockdown or overexpression on PC3 cell proliferation. (**C**) The effect of down-regulation or overexpression of circ_0001671 on an apoptosis-related gene by western blot. (**D**) The effect of circ_0001671 knockdown or overexpression on PC3 cell proliferation was assessed by wound healing. (**E**) The effect of circ_0001671 knockdown or overexpression on PC3 cell proliferation was assessed by EdU. (**F**) The effect of circ_0001671 knockdown or overexpression on the cycle-related gene by western blot. (**G**) The effect of circ_0001671 knockdown or overexpression on the migration and invasion of PC3 cells was assessed by transwell. *p < 0.05, **p < 0.01, by Student’s t-test. Error bars represent SD.

To investigate the regulatory effect of circ_0001671 on cell proliferation, multiple assays were conducted, including Cell Counting Kit-8 (CCK8), 5-ethynyl-20-deoxyuridine (EdU), wound healing, and transwell assays. CCK8 analysis revealed a significant increase in the proliferation of PC3 cells upon overexpression of circ_0001671 (Fig. 3B). Congruent findings were obtained from wound healing, migration, invasion, and EdU assays (Fig. 3D, F, and G). Western blot assays further indicated that overexpression of circ_0001671 upregulated the expression of proliferation-related gene PCNA, as well as cell cycle-related genes CDK2, CDK6, cyclin D1, and cyclin E1.

In addition, the role of circ_0001671 in regulating PC3 cell apoptosis was examined. Western blot analysis showed that circ_0001671 overexpression led to an upregulation of the apoptosis-related gene Bcl-2 and a downregulation of Bax (Fig. 3A, C, and E). These effects were reversed upon knockdown of circ_0001671.

### Circ_0001671 serves as a sponge for miR-27b-3p

**Figure 4:**
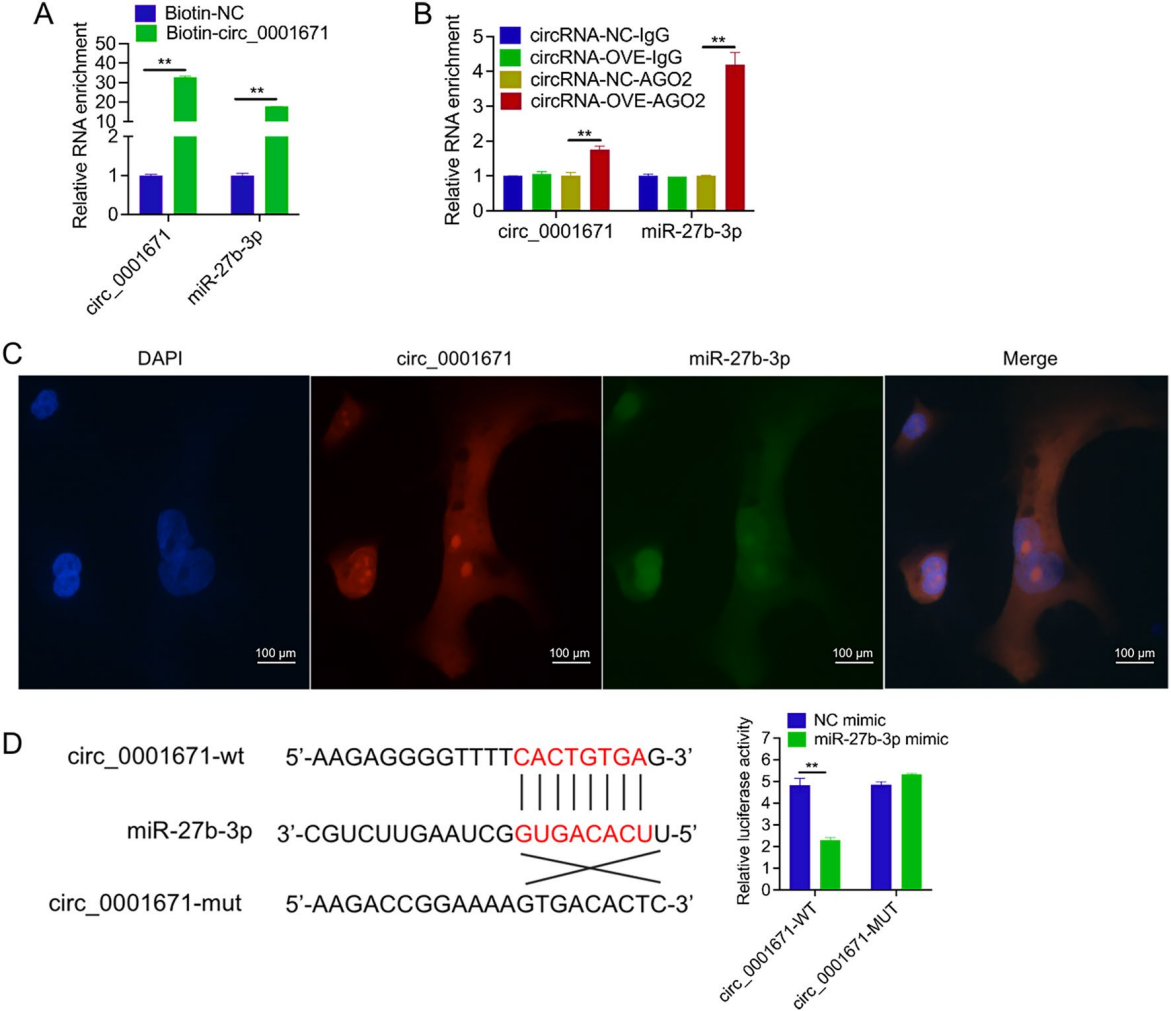
Circ_0001671 serves as a sponge for miR-27b-3p. (**A**) Biotin circRNA pull-down assay using biotin-circ_0001671, compared to control biotin-NC. (**B**) RIP analysis using anti-Ago2 antibody followed by detection of circ_0001671 and miR-27b-3p. Values were normalized to background RIP levels detected by IgG isotype controls. (**C**) Co-localization of circ_0001671 and miR-27b-3p in PC3 cells was assessed by FISH. Scale bars are 100 μm. (**D**) The luciferase activity of PC3 cells was measured after co-transfection with either the wild-type (WT) or mutant (MUT) circ_0001671 luciferase reporter vector and miR-27b-3p mimics or control miR mimics.

In the current study, further exploration was conducted to understand the molecular mechanisms underlying the effects of circ_0001671 on prostate cancer cells. Earlier studies have suggested that circRNAs can function as miRNA sponges, depending on their subcellular localization. Cytoplasmic localization of circ_0001671 was confirmed in PC3 cells (Fig. 1K), leading to the hypothesis that circ_0001671 may function by sponging miRNAs. Subsequently, a pull-down assay using biotin-labeled probes targeting the circ_0001671 junction site was conducted, followed by qPCR. A notable increase in the abundance of miR-27b-3p was observed compared to the control (Fig. 4A). Considering that miRNAs can inhibit translation and degrade mRNA in an Ago2-dependent manner, an anti-Ago2 RNA immunoprecipitation (RIP) assay was performed. The assay demonstrated significant enrichment of both circ_0001671 and miR-27b-3p in the anti-Ago2 group compared to the anti-IgG group (Fig. 4B), suggesting a regulatory role for circ_0001671 in prostate cancer.

To validate the sponging ability of circ_0001671 for miR-27b-3p, we conducted FISH co-localization and luciferase experiments, luciferase constructs containing potential target sites were generated, along with mutant constructs featuring altered miR-27b-3p binding sites. FISH results showed that cell colocalization existed in circ_0001671 and miR-27b-3p (Fig. 4C). The luciferase activity of the wild-type reporter was significantly reduced upon overexpression of miR-27b-3p through miRNA mimics, while the mutant reporters remained unaffected (Fig. 4D). Collectively, these results confirm that circ_0001671 serves as a miR-27b-3p sponge in prostate cancer cells.

### Circ_0001671 promotes PC3 cells proliferation via the miR-27b-3p/BLM *axis*

**Figure 5:**
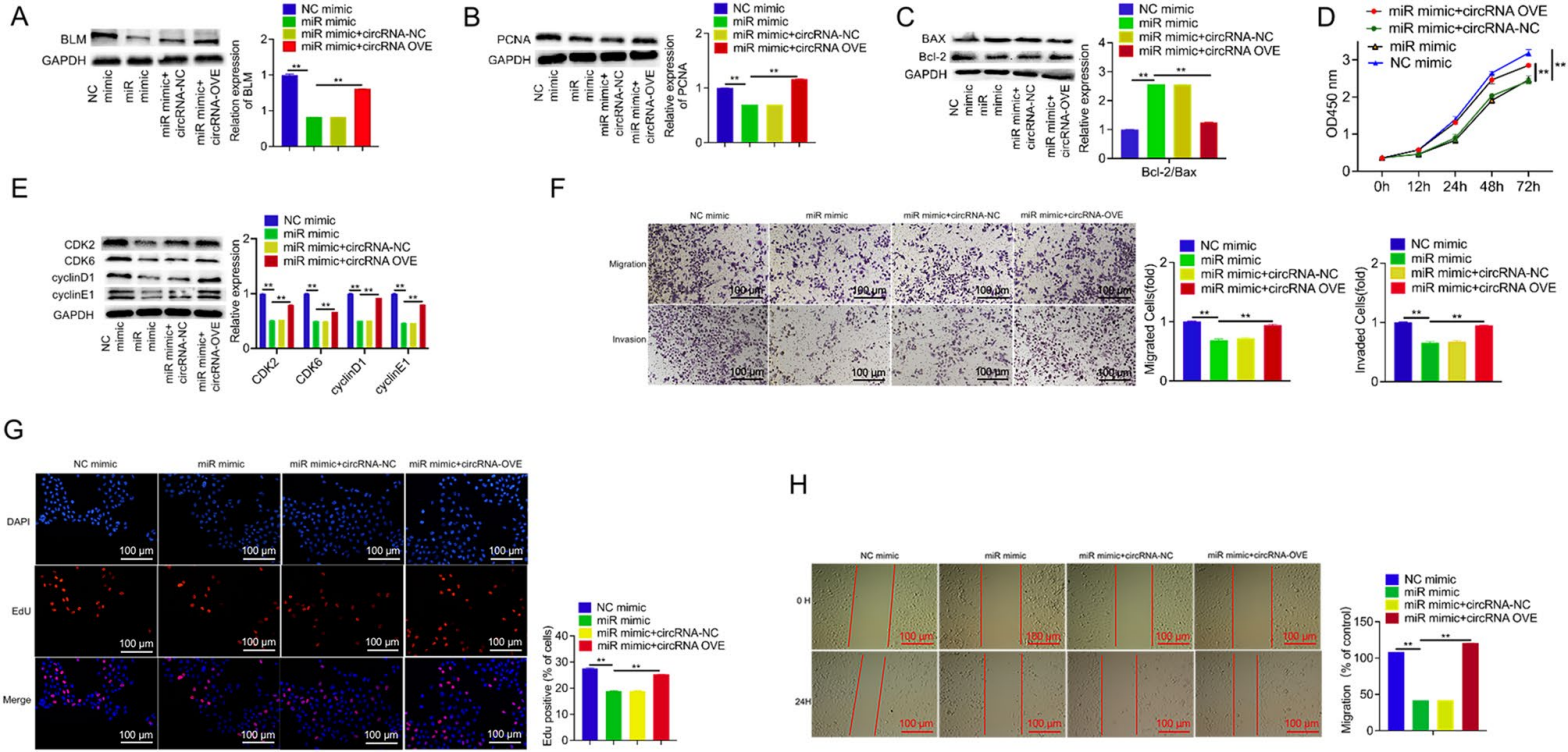
Circ_0001671 exerts biological functions in PCa through the miR-27b-3p/BLM axis. PC3 cells were transfected with miR-27b-3p mimic or a combination of circ_0001671 vector and miR-27b-3p mimic. Western blot detection of BLM (**A**), proliferation-related genes (**B**), apoptosis-related genes (**C**), and cycle-related genes protein levels (**E**). In vitro cell proliferation was detected by CCK8 assay (**D**), EdU (**G**), wound healing (**H**), and migration and invasion assays (**F**). *p < 0.05, **p < 0.01, by Student’s t-test. Error bars represent SD.

To elucidate whether circ_0001671 operates through the miR-27b-3p/BLM axis, a rescue assay was executed. Data indicated that overexpression of miR-27b-3p led to a marked reduction in BLM expression levels in PC3 cells (Fig. 5A). This effect was reversed when miR-27b-3p and circ_0001671 were co-overexpressed, as evidenced by western blot analysis (Fig. 5A).

Subsequent CCK8 and EdU assays exhibited a substantial decline in PC3 cell proliferation upon miR-27b-3p overexpression (Fig. 5D and G). Consistent results were obtained from the wound healing, migration, and invasion assays (Fig. 5H and F). Additionally, western blot assays revealed that miR-27b-3p overexpression led to decreased expression levels of the proliferation-related gene PCNA and cycle-related genes CDK2, CDK6, cyclinD1, and cyclinE1. An accompanying reduction in the expression of the apoptosis-related gene Bcl-2 and an increase in Bax expression were observed. However, these effects were reversed upon co-overexpression of miR-27b-3p and circ_0001671 (Fig. 5B, C, and E). Collectively, these findings substantiate the hypothesis that circ_0001671 augments PC3 cell proliferation by sponging miR-27b-3p and operating through the circ_0001671/miR-27b-3p/BLM regulatory axis.

### In vivo relevance of the Circ_0001671/miR-27b-3p/BLM *axis* in PCa progression

**Figure 6:**
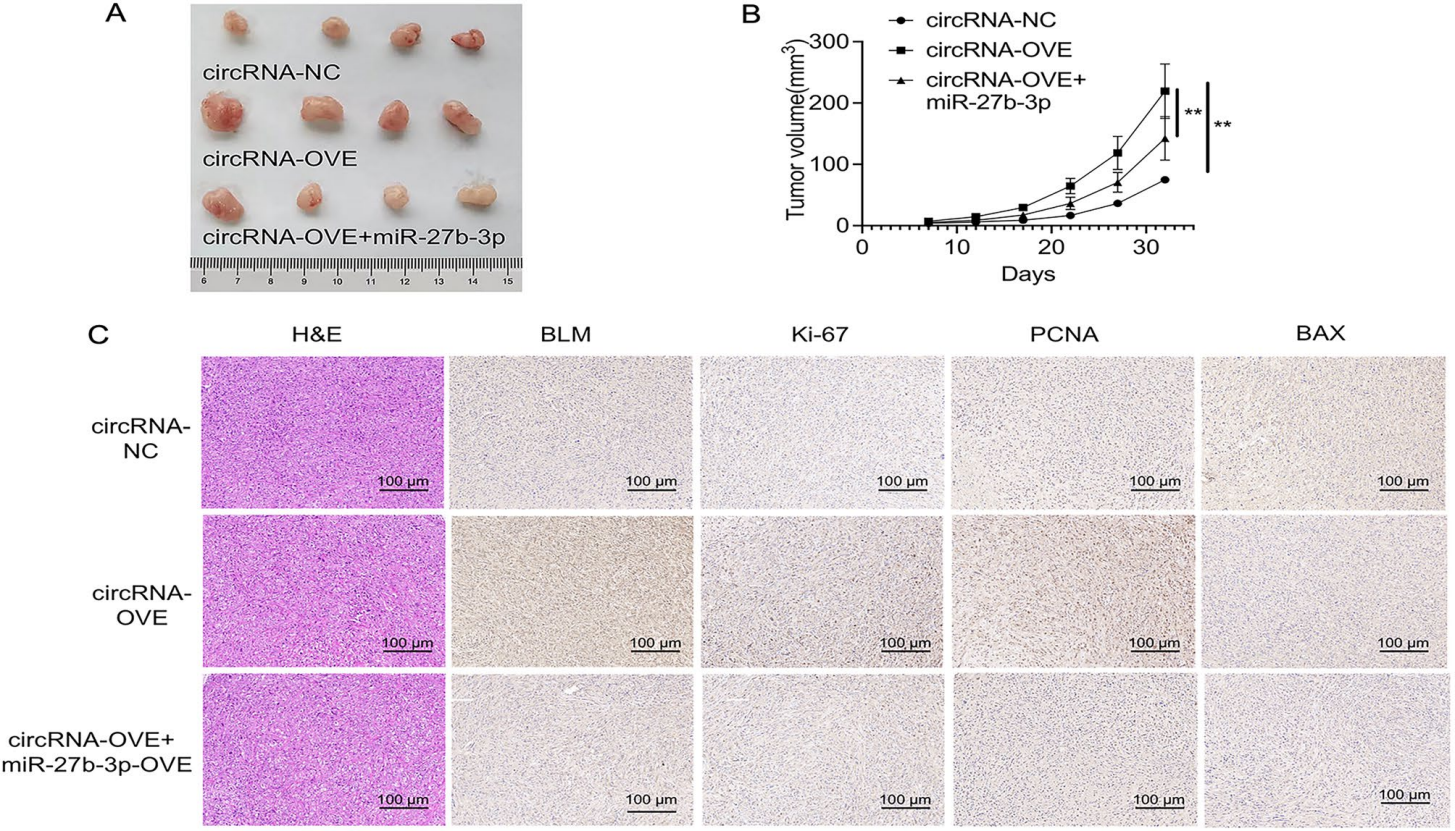
The circ_0001671/miR-27b-3p/BLM Axis Is Important for PCa Progress In Vivo. (**A**) Images of a given set of tumors (n = 4). (**B**) Growth curves of tumors in nude mice after the indicated treatments. (**C**) H&E and IHC staining of BLM, Ki-67, PCNA, and BAX in the indicated treated tumors. Scare bars, 100 μm. *p < 0.05, **p < 0.01, by Student’s t-test. Error bars represent SD.

In order to verify the oncogenic capacity of circ_0001671 via the miR-27b-3p/BLM axis in an in vivo context, a xenograft tumor model was established in nude mice. The research reveals that the overexpression of circ_0001671 leads to accelerated tumor growth, characterized by notably elevated tumor activity in the overexpression group relative to the control group. This effect, however, was observed to be partially counteracted by the heightened expression of miR-27b-3p (Fig. 6A, B). Through immunohistochemical assay, elevated levels of BLM, Ki-67, and PCNA were observed in the tumor tissues with circ_0001671 overexpression when compared to those in the control group. Conversely, the level of Bax was found to be diminished. Moreover, the overexpression of miR-27b-3p was seen to partially mitigate these changes (Fig. 6C). These results provide evidence for the significant role of circ_0001671 in the progression of prostate cancer (PCa) via the miR-27b-3p/BLM axis in an in vivo environment.

## Discussion

Abnormal expression of BLM helicase has been observed in a range of cancer types, notably exhibiting elevated levels in prostate cancer (PCa). Although considered a significant factor for PCa treatment, the underlying regulatory mechanisms governing BLM expression remain elusive. The present study addresses this gap in understanding by identifying a highly stable and abundant circRNA, designated as circ_0001671, in PCa specimens.Quantitative PCR analyses conducted on both tissues and cellular samples have revealed a marked upregulation of circ_0001671 in PCa tissues and cells. Functionally, circ_0001671 acts as an RNA sponge for miR-27b-3p, which in turn results in elevated BLM expression. Through gain and loss-of-function studies, it was ascertained that circ_0001671 plays a significant role in enhancing cellular proliferation, migration, and invasion while attenuating apoptosis. Further support for this mechanism is provided by both in vitro and in vivo experiments, where a facilitative role for circ_0001671 in tumor growth was clearly evidenced.

Initially, circular RNAs were considered mere byproducts of aberrant RNA splicing. However, accumulating evidence has shifted this perspective, indicating that circular RNAs are prevalent across various human cell types and have been conserved throughout evolutionary history. Consequently, the classification of circular RNAs as waste products is undergoing reevaluation. Specifically, a substantial number of these circular RNAs have been identified and are posited to play pivotal roles in the onset and progression of diverse cancer types (Chen^9,11,13^). Additional functionalities attributed to circular RNAs have been elucidated, encompassing roles such as serving as scaffolds in the assembly of protein complexes, sequestering proteins away from their native subcellular locations, modulating the expression levels of parent genes, and governing alternative splicing as well as RNA–protein interactions. Moreover, certain circular RNAs act as sponges for microRNA (miRNA). A multitude of studies have furnished strong evidence regarding the involvement of circular RNAs (circRNAs) in cancer progression through various molecular pathways. For instance, For example, circRNA_101237 has been observed to advance non-small cell lung cancer (NSCLC) via the miRNA-490-3p/MAPK1 axis^26^. Analogously, hsa_circRNA_104348 has been implicated in the progression of hepatocellular carcinoma by modulating the miR-187-3p/RTKN2 axis and activating the Wnt/β-catenin pathway^27^. Other noteworthy instances include circRNA_100367, which regulates radiation sensitivity in esophageal squamous cell carcinomas via the miR-217/Wnt3 pathway^28,29^. Additional examples include hsa_circ_001783, which impacts breast cancer progression by sponging miR-200c-3p^30^, circCAMSAP1, which enhances tumor growth in colorectal cancer through the miR-328-5p/E2F1 Axis^31^, circEHBP1, which contributes to lymphangiogenesis and lymphatic metastasis in bladder cancer via miR-130a-3p/TGFβR1/VEGF-D signaling^5,6^, and circRNA.33186, which is implicated in the pathogenesis of osteoarthritis by sponging miR-127-5p^32^.

MiRNA is predominantly implicated in the suppression of mRNA expression by targeting the 3' UTR of specific genes. One such miRNA, MiR-27b-3p, has been identified as a potential tumor suppressor across various cancer types^33^. Investigations have demonstrated that MiR-27b-3p exerts a negative regulatory effect on DNA repair and exhibits diminished expression in lung cancer tissues^34^. Moreover, it was found to exert anti-tumoral effects by inhibiting the target gene Fzd7. Conversely, overexpression of MARCH 7 in endometrial cancer cells has been found to promote cell invasion and metastasis, and miR-27b-3p has been shown to reduce the stimulating effect induced by MARCH 7^28,29^. Additionally, a significant inhibition of cell proliferation in gastric cancer was attributed to miR-27b-3p^35^, which also inhibited the proliferation, migration, and invasion of colorectal cancer cells^36^. Prior studies indicated that miR-27b-3p could directly target the BLM gene 3' UTR, thereby suppressing the proliferation, migration, and invasion of prostate cancer cells^8,25^. However, it had not been previously reported that circRNA could influence BLM expression by serving as a miR-27b-3p sponge. To investigate this, MiR-27b-3p was employed as a bridge to screen for circRNAs that could potentially regulate BLM expression. In the current study, circ_0001671 was identified as a highly expressed oncogene in prostate cancer (PCa) cells. Moreover, a positive correlation was observed between the expression trends of circ_0001671 and BLM. This stable circular transcript displayed resistance to RNase R digestion. Subsequent observations indicated that circ_0001671 is localized in the cytoplasm, suggesting its role as a potential miRNA sponge. This proposition was substantiated by bioinformatics analyses confirming an interaction between circ_0001671 and miR-27b-3p. Further, luciferase activity assays revealed a negative correlation between the expression levels of circ_0001671 and miR-27b-3p.

To deepen the understanding of this interaction, biotin-labeled probes and RNA immunoprecipitation (RIP) assays were conducted. These facilitated the pull-down of circ_0001671 and allowed for the subsequent detection of its expression level, as well as that of miR-27b-3p, via qPCR. In conclusion, the findings reveal that miR-27b-3p binds to circ_0001671, as corroborated by its co-pull-down with circ_0001671, suggesting that circ_0001671 functions as a sponge targeting miR-27b-3p directly.

In prostate cancer cells, overexpression of circ_0001671 has been observed to result in elevated BLM expression. Contrarily, such an effect on BLM by circ_0001671 can be negated upon overexpression of miR-27b-3p. Furthermore, experimental data revealed that overexpression of circ_0001671 promotes cell proliferation, migration, and invasion while inhibiting apoptosis. In vivo studies confirmed that circ_0001671 overexpression significantly enhances the formation of subcutaneous tumors in nude mice. In contrast, miR-27b-3p overexpression was found to suppress tumor formation, an effect that could be negated by the overexpression of circ_0001671.The findings underscore the critical role of circ_0001671 in prostate cancer progression. To the researchers' awareness, this constitutes the inaugural study elucidating the mechanism by which BLM is regulated by circ_0001671 in prostate cancer development. The results point toward the potential of circ_0001671 serving as a molecular target for therapeutic intervention in prostate cancer.

## Materials and methods

### Cell lines and tissue

Sixteen pairs of carcinoma and adjacent tissues were collected from Zunyi Medical University according to ARRIVE guidelines. Ethical approval for this work was granted by the Institutional Review Board of Zunyi Medical University. Human prostate cancer cell lines, including PC3, 22RV1, and LNCaP, as well as human normal prostate epithelial RWPE-1 cells, were obtained from Zhong Qiao Xin Zhou Biotechnology (Shanghai, China).

### Cell culture and transfection

PC3 cells were maintained in DMEM-F12 medium (Gibco, NY, USA) enriched with 10% fetal bovine serum (VivaCell, China) and supplemented with 1% streptomycin-penicillin (Gibco, NY, USA). In a similar fashion, 22RV1 and LNCaP cells were cultured in RPMI-1640 medium (Gibco, NY, USA) containing 10% fetal bovine serum (VivaCell, China) and 1% penicillin–streptomycin (Gibco, NY, USA). RWPE-1 cells were sustained in a specialized medium for RWPE-1 cells (ZQ-1303, ZQXZ Bio, Shanghai, China). All four cell types were incubated at 37 °C in a humidified atmosphere containing 5% CO_2_.Small interfering RNA (siRNA) targeting circ_0001671 and vectors designed to elevate miRNA expression levels (miRNA-mimic) were synthesized by GenePharma (Shanghai, China). Vectors to augment circRNA expression (over-circRNA) were engineered by Tsingke (Chongqing, China). The lentivirus vector was acquired from Shanghai Genechem (Shanghai, China). Transfection was performed using specific vectors (si-circRNA, over-circRNA, miRNA mimic at a concentration of 100 nM) and FuGENE HD (Promega, Madison, WI, USA), as per the manufacturer's guidelines. Following transfection, cells were utilized in subsequent experiments.

### CircRNA screening

In order to identify circRNAs that might interact with miR-27b-3p, a selection of tools was employed. These tools include circBank (http://www.circbank.cn/index.html), ENCORI (http://starbase.sysu.edu.cn/index.php), and a peer-reviewed article^37^. These resources were utilized to rigorously screen for potential circRNA candidates that could associate with miR-27b-3p.

### qRT-PCR

Total RNA from PC3, 22RV1, LNCaP, and RWPE-1 cells was extracted utilizing Trizol separation reagents (Invitrogen, Carlsbad, CA, USA), in accordance with the manufacturer's protocol. The quality of the RNA samples was assessed through ultraviolet spectrophotometry (Invitrogen, Carlsbad, CA, USA).For the reverse transcription of miRNAs, circRNA, and BLM, designed stem-loop primers, divergent primers, and general primers were employed, respectively. A total of 2.5 µg of RNA was used for each reverse transcription, which was facilitated by the RevertAid First Strand cDNA Synthesis Kit (Invitrogen, Carlsbad, CA, USA).Quantitative RT-PCR assays were conducted using CFX-96 Real-Time PCR Systems and SYBR Green Mix (Bio-Rad Laboratories, CA, USA). Each sample was assessed in triplicate. GAPDH served as the internal control for evaluating circ_0001671 and related mRNA expression levels, while U6 was used as the internal control for miR-27b-3p expression. Data were analyzed by calculating the 2^–ΔΔCq^^38^ relative fold change method. The specific primers employed for real-time PCR are delineated in Table S1.

### Immunofluorescence staining

PC3 cells were seeded in a six-well plate and incubated for 48 h. Cells were then fixed using 3.7% paraformaldehyde and permeabilized with 0.25% Triton X-100. Subsequently, blocking was carried out with 3% Bovine Serum Albumin (BSA). Cells were incubated with a primary antibody on a shaking table at 4 °C overnight. This was followed by incubation with a Cy3-conjugated secondary antibody at room temperature for 2 h. The primary antibody utilized was anti-BLM (dilution ratio of 1:150; Bioss, China), and the secondary antibody was fluorescent Cy3-conjugated goat anti-rabbit antibodies (dilution ratio of 1:600; Invitrogen, Carlsbad, CA, USA). Fluorescence imaging was conducted using a fluorescence microscope (Nikon, Japan).

### Western blot analysis

Total proteins were extracted from cells utilizing Radioimmunoprecipitation Assay (RIPA) lysis buffer (Solarbio, China). Protein concentrations were then ascertained through the Bicinchoninic Acid (BCA) protein assay (Solarbio, China). Electrophoresis was performed on SDS–polyacrylamide gels, followed by transfer to PVDF membranes (Millipore, Carrigtwohill, Ireland). The membranes were then blocked with skim milk and incubated overnight at 4 °C with primary antibodies. After washing, a secondary antibody was applied and incubated at room temperature for 2 h. The antibodies used were anti-BLM (1:900 dilution; Bios, China), anti-Bax (1:5,000 dilution; Proteintech, USA), anti-PCNA (1:3,000 dilution; Proteintech, USA), anti-CDK6 (1:5,000 dilution; Proteintech, USA), anti-Cyclin D1 (1:5,000 dilution; Proteintech, USA), anti-Bcl-2 (1:2000 dilution; Proteintech, USA), anti-Cyclin E1 (1:1000 dilution; Proteintech, USA), anti-CDK2 (1:1000 dilution; Proteintech, USA), GAPDH (1:1000 dilution; Proteintech, USA), and goat-anti-rabbit, goat-anti-mouse secondary antibody (1:1,0000 dilution; Proteintech, USA). Subsequently, the results were quantified and images were processed using ImageJ software.

### Immunohistochemistry

Immunohistochemistry (IHC) was conducted on mouse tumor tissue sections as previously cited in relevant literature^25^. The primary antibody was consistent with those used in Western blot analyses. Tissue sections were incubated overnight at 4 °C with the primary antibody and subsequently with the secondary antibody. Diaminobenzidine (DAB) was employed as the chromogen, and nuclei were counterstained with hematoxylin.

### Nuclear-cytoplasmic separation

Nuclear and cytoplasmic RNA fractions were isolated using the Cytoplasmic and Nuclear RNA Purification Kit (Norgen Biotek, Thorold, ON, CAN), following the manufacturer's guidelines. Specifically, PC3 cells underwent lysis with Lysis Buffer J. Nuclear and cytoplasmic components were then separated by centrifugation. The supernatant was transferred to a new RNase-free tube, and the residual lysate was washed with cell separation buffer and subsequently centrifuged. The lysate and supernatant were then combined with Buffer SK, and an equal volume of ethanol was passed through a filter element. The sample was washed with Wash Solution A, and finally, both cytoplasmic and nuclear RNA were eluted with Elution Buffer E.

### Luciferase reporter assay

The luciferase reporter construct for circ_0001671 was synthesized by GenePharma (Shanghai, China). This construct was then transfected into PC3 cells along with 100 nM miRNA mimics and a control plasmid. After 48 h of incubation, the Dual-Luciferase Reporter System (Promega, Madison, WI, USA) was employed to quantify both firefly and Renilla luciferase activities.

### Cell proliferation assay

To evaluate the influence of circ_0001671 on the proliferation of PC3 cells, several methods were employed, including scratch assay, CCK8 (APExBIO, USA), and EdU (APExBIO, USA). In the scratch assay, cells transfected to 95% confluency were scratched using a pipette tip. After 24 h, the healing distance was measured to assess cell migration capabilities. For CCK8 assays, cells were transfected with either vector or siRNA for 48 h, followed by seeding in 96-well plates at a density of 2 × 10^3^ cells per well. Cells were incubated for for 2 h at varying time periods (12 h, 24 h, 48 h, or 72 h), and cell proliferation was evaluated by measuring absorbance at 450 nm using a Spectra Max 250 spectrophotometer (BioTek, USA). Each experiment was performed in triplicate. In the EdU assay, cells transfected with vector or siRNA were seeded in 24-well plates at a density of 2 × 10^5^ cells per well. Following incubation with 20 µM EdU for 2 h, cells were fixed with 3.7% formaldehyde and permeabilized with 0.25% Triton X-100. After washing with PBS, cells were incubated with Click Buffer for 30 min at room temperature. Hoechst 33,258 staining was employed to visualize nuclei. EdU uptake rates were calculated as the ratio of EdU-positive cells to Hoechst 33,258-positive cells.

### FISH assay

In accordance with the manufacturer's instructions, a FISH assay was carried out using a kit from GenePharma (Shanghai, China) to ascertain the localization of circ_0001671. Cy3-labeled circ_0001671 probes were hybridized with FAM-labeled miR-27b-3p probes, also obtained from GenePharma. PC3 cells were fixed in 3.7% paraformaldehyde and permeabilized with 0.1% buffer A for 20 min. Subsequently, the cells were incubated in sealing buffer at 37 °C for 40 min and washed with 2 × buffer C. Hybridization with circ_0001671 and miR-27b-3p probe mixes was performed overnight. The slides underwent washing steps involving 0.1% buffer F at 37 °C for 12 min, 2 × buffer C at 60 °C for 12 min, and 2 × buffer C at 37 °C for 10 min. Finally, 4’,6-diamidino-2’-phenylindole (DAPI) was added and incubation occurred at 35 °C for 15 min. Confocal microscopy was utilized for result analysis. Probe sequences are available in Table S2.

### CircRNA pull-down

Biotin-labeled circ_0001671 probes and control probes were synthesized by Huada Gene (Guangdong, China). For this assay, an RNA–protein pull-down kit (Invitrogen, Carlsbad, CA, USA) was utilized. Streptavidin-coated magnetic beads were prepared and washed with an equal volume of 20 mM Tris (pH 7.5), followed by the addition of an equal volume of 1 × RNA capture buffer. These beads were then incubated with 50 pmol of labeled RNA at 35 °C for 35 min with agitation. Subsequently, the beads were incubated overnight with cell lysates generated using standard lysis buffer. Quantitative real-time RT-PCR was executed to quantify the expression of target genes. Probe sequences used for this analysis can be found in Table S3.

### RNA immunoprecipitation

For the Ago2 RIP assay, anti-Ago2 antibodies (Proteintech, USA) and anti-IgG antibodies (Millipore, Merck, Germany) were employed. The Magna RIP RNA-binding protein immunoprecipitation kit (Millipore, Merck, Germany) was used, following the manufacturer's protocol. Subsequent to the immunoprecipitation, real-time RT-PCR was performed to quantify mRNA expression.

### RNase R treatment

In the case of RNase R treatment, 2 µg of extracted RNA was incubated either with or without RNase R (Epicenter Technologies, Madison, WI, USA) for 15 min at 37 °C. Following incubation, quantitative real-time RT-PCR was conducted to evaluate gene expression levels.

### Migration and invasion assay

Following 24 h of transfection, 1.5 × 10^5^ PC3 cells were suspended in 180 µL of serum-free medium and seeded into the upper chambers of a transwell system (pore size 8 µm, Costar). For invasion assays, Matrigel was applied to the upper chambers, while for migration assays it was omitted. The lower chambers were filled with 600 µL of complete growth medium containing 20% fetal bovine serum to act as a chemoattractant for PC3 cells. The cells were then incubated for 24 h at 37 °C and 5% CO2. Post incubation, cells remaining in the upper chamber were removed using a cotton swab, while the cells that had migrated or invaded were fixed with 3.7% paraformaldehyde and stained with Giemsa. Microscopic examination was subsequently carried out (Nikon, Japan).

### Xenograft tumor model

Animal experiments were conducted with approval from the Committee for Animal Care and Use at Guizhou University according to the Basel Declaration. BALB/c nude mice, aged 5 weeks, were randomly assigned into three groups (n = 4 per group): PC3 cells with control over-vector (circRNA-NC), PC3 cells with circ_0001671 Vector (circRNA-OVE), and PC3 cells with miR-27b-3p Vector and circ_0001671 Vector (circRNA-OVE + miR-27b-3p). Cells were subcutaneously injected into the mice at a concentration of 4 × 10^6^ cells in 0.2 mL of PBS per mouse. Tumor volumes were monitored every 5 days using an electronic vernier caliper. At the end of the 4-week period, the animals were euthanized, and tumor volumes were again measured. Collected tumor tissues were subjected to staining procedures including hematoxylin and eosin (HE), and immunohistochemistry (IHC).

### Statistical analysis

Statistical data were presented as mean ± SD and were derived from independent experiments performed in triplicate. The software GraphPad Prism 9 (GraphPad, La Jolla, CA, USA) was utilized for all statistical analyses. A two-tailed Student’s t-test was employed to determine the statistical significance between two datasets. A *p*-value of less than 0.05 was considered to indicate statistical significance.

### Ethics approval and consent to participate

The Ethics Committee of Affiliated Hospital of Zunyi Medical University approved the study protocol in accordance [Compliance Medical Ethics Review (2021) no. 1–037. Before enrollment, all participants provided written informed consent. Additionally, the Committee for Animal Care and Use of Guizhou University approved the animal study protocol (EAE-Gzu-2022-E022). we confirming the study is reported in accordance with ARRIVE guidelines.

### Supplementary Information

Supplementary Information 1.

Supplementary Information 2.

Supplementary Information 3.

## Data Availability

The datasets used and/or analysed during the current study available from the corresponding author on reasonable request.
