# Supplementary material for: Circ_0001671 regulates prostate cancer progression through miR-27b-3p/BLM axis
Source: Sci Rep. 2024 May 28;14:12181. doi: 10.1038/s41598-024-63068-x (PMC11133351; doi:10.1038/s41598-024-63068-x)
Supplement: Supplementary file 1 — Supplementary Information 1. [file 41598_2024_63068_MOESM1_ESM.docx]

| Table S1 qPCR primers | | | |
| --- | --- | --- | --- |
| miR-27-3p (5’-3’) | R | CGGGCTCTCTGTTCACAGTGG | 76bp |
|  | F | CAAAGCAGGGTCCGAGGTATC |  |
| circ_0001671  (Divergent) | R | TTGATGAAAGGTAGCCAGGTAGGTG | \| 72bp \| \| --- \| |
|  | F | TATTTTTAAAGATGGACAGAGACGT |  |
| circ_0001671  (Convergent) | R | CAGTGAAAACCCCTCTTCTTGTGCC | \| 97bp \| \| --- \| |
|  | F | TGGTGACCTTTAGCCCCCTGATGGA |  |
| BLM | R | GAAGAACTATCACCCCCCAGC | 245bp |
|  | F | AAGCGACATCAGGAGCCAAT |  |
| GAPDH | R | CCACCACCCTGTTGCTGT | 104bp |
|  | F | CTCCTCCACCTTTGACGC |  |
| U6 | R | AACGCTTCACGAATTTGCGT | 93bp |
|  | F | CTCGCTTCGGCAGCACA |  |
