# Supplementary material for: Circ_0001671 regulates prostate cancer progression through miR-27b-3p/BLM axis
Source: Sci Rep. 2024 May 28;14:12181. doi: 10.1038/s41598-024-63068-x (PMC11133351; doi:10.1038/s41598-024-63068-x)
Supplement: Supplementary file 3 — Supplementary Information 3. [file 41598_2024_63068_MOESM3_ESM.docx]

| Table S3 Biotin probe sequence | |
| --- | --- |
| Biotin-circ_0001671 | TCTCTGTCCATCTTTAAAAATAGGCCAATGGGC |
| Biotin-NC | GCCCATTGGCCTATTTTTAAAGATGGACAGAGA |
